# Anaphylactic Shock Due to Technetium (99mTc)-Tetrofosmin During Myocardial Perfusion Scintigraphy: A Case Report

**DOI:** 10.7759/cureus.52644

**Published:** 2024-01-20

**Authors:** Sho Hashimoto, Tetsuya Tanaka, Yoshiaki Shimoda, Mariko Tanaka, Morihiko Kondo

**Affiliations:** 1 Cardiology, Japan Community Healthcare Organization (JCHO) Kobe Central Hospital, Kobe, JPN

**Keywords:** allergic reaction, technetium tc-99m, myocardial scintigraphy, stable ischemic heart disease, anaphylactic shock

## Abstract

Myocardial perfusion scintigraphy is a popular minimally invasive method for evaluating chronic coronary disease (CCD). We performed myocardial scintigraphy to assess CCD in a 74-year-old man with a history of allergy to contrast media. The patient developed anaphylactic shock immediately after the administration of the technetium (^99m^Tc)-tetrofosmin preparation. This is the first report of anaphylactic shock due to ^99m^Tc-tetrofosmin administration during myocardial perfusion scintigraphy.

## Introduction

Coronary computed tomography angiography (CCTA) and myocardial perfusion scintigraphy are recommended and widely performed as minimally invasive evaluation methods for chronic coronary disease (CCD) [1, 2]. The frequency of side effects from iodine contrast media used in CCTA has been reported to be at least 0.7% [3], with the most significant risk factor being a history of contrast media allergy [4]. In patients at high risk for allergy to contrast medium, myocardial perfusion scintigraphy is especially desirable for evaluating CCD. Technetium (^99m^Tc)-tetrofosmin is widely used for myocardial perfusion scintigraphy [5]. Here, we report the first case of anaphylactic shock due to ^99m^Tc-tetrofosmin administration during myocardial perfusion scintigraphy.

## Case presentation

**Figure 1:**
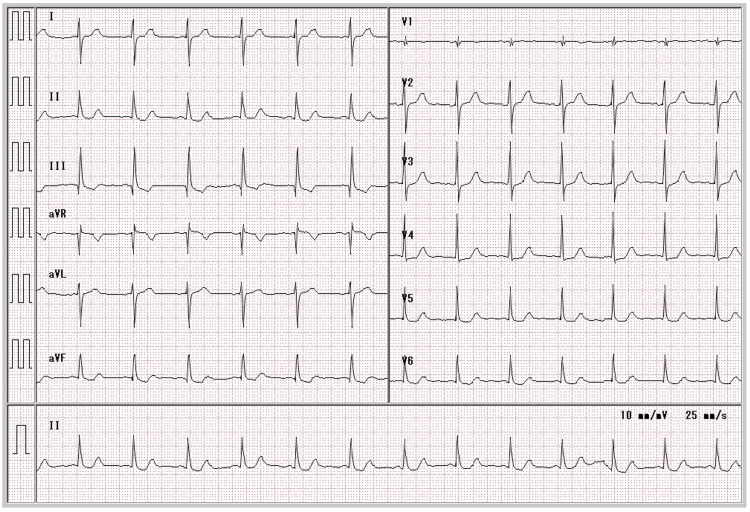
Initial electrocardiogram at rest showing horizontal ST-segment depression at V4–6.

A 74-year-old male with a history of hypertension, interstitial pneumonia, and anaphylactic shock due to contrast media use more than 30 years prior was referred to our hospital due to an abnormality on routine electrocardiography without chest pain. On physical examination, his vitals were stable, and both his heart and respiratory sounds were normal. The electrocardiogram at rest showed a sinus rhythm and horizontal ST-segment depression at V4-6 (Figure 1).

Echocardiography revealed no regional wall motion abnormalities. We performed adenosine stress myocardial perfusion scintigraphy to evaluate CCD and administered a continuous intravenous infusion of 120 μg/kg of adenosine for six minutes, accompanied by an intravenous injection of 296 MBq of ^99m^Tc-tetrofosmin preparation.

A few minutes later, the patient presented with wheals and itching of the face, chest, and upper extremities. His blood pressure was 113/62 mmHg, and his pulse rate was 92 beats per minute without dyspnea. The symptoms quickly abated with a 200-mg hydrocortisone infusion. We attributed these symptoms to allergic reactions to adenosine and acquired the first image 40 minutes later, as scheduled.

Subsequently, we administered a second intravenous dose of 740 MBq of ^99m^Tc-tetrofosmin preparation three hours later in the radioisotope examination room. Immediately after the injection, the patient experienced moodiness, cold sweats, chest discomfort, systemic swelling, erythema, and wheals. The patient had a systolic blood pressure of 75 mmHg and a pulse rate of 82 beats per minute, without dyspnea. We placed him in a supine position, elevated his lower extremities, rapidly rehydrated him, and transferred him to the emergency room. His vitals improved upon reaching the emergency room; his blood pressure was 123/61 mmHg, and his pulse rate was 85 beats/min. He was intravenously administered 200 mg of hydrocortisone and 25 mg of hydroxyzine, and the symptoms improved drastically.

We then retransferred him to the radioisotope examination room and acquired a second image 40 minutes later, as scheduled. A blood examination in the emergency room showed no specific abnormalities. The patient was discharged the next day after confirming the absence of flare-up signs.

**Figure 2:**
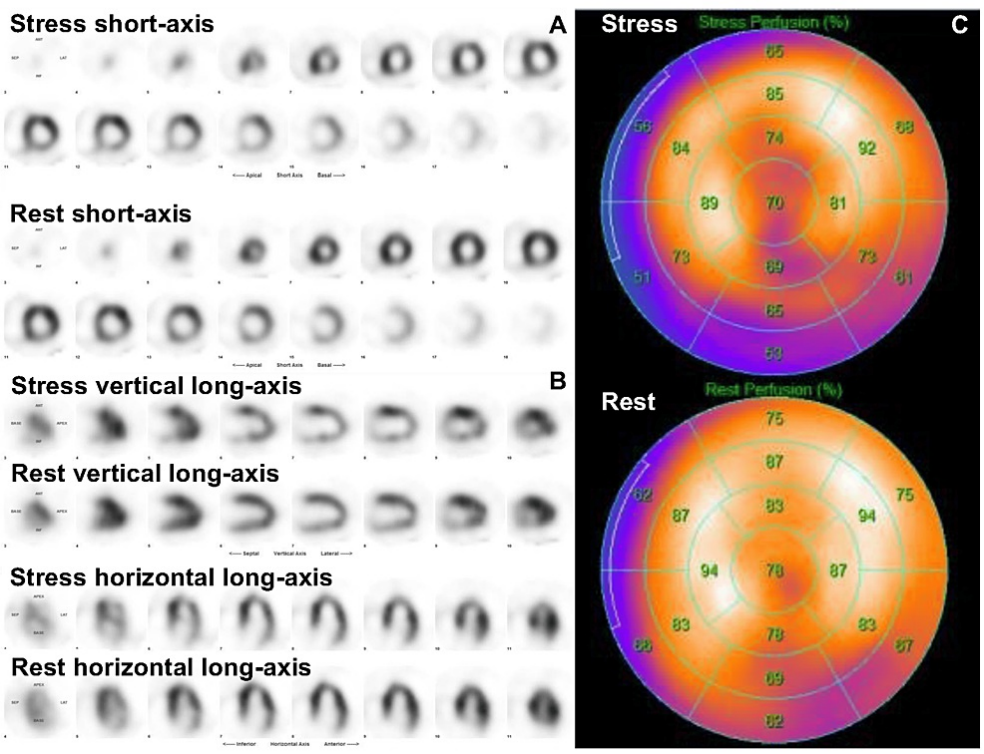
Results of myocardial perfusion scintigraphy Stress images show moderately reduced uptake in the inferior wall and mildly reduced uptake in the anterior and apex walls. Rest images show fill-in in the inferior, anterior, and apex walls. A) Short-axis images of the left ventricle at stress (the top two rows) and at rest (the bottom two rows). B) Vertical (the top two rows) and horizontal (the bottom two rows) long-axis images of the left ventricle at stress (upper) and at rest (lower). C) Bull’s-eye map at stress (upper) and at rest (lower).

We then referred him to the university hospital, where he had been followed for interstitial pneumonia, at the patient’s request. Myocardial perfusion scintigraphy indicated decreased uptake at stress and fill-in at rest in the inferior, anterior, and apex walls (Figure 2), suggesting myocardial ischemia.

As the patient was symptomatically stable and had a history of anaphylactic shock due to contrast media, he was only followed up with beta-blockers, aspirin, and statins without undergoing a coronary angiogram. The patient was referred from the university hospital to his primary care doctor and is currently stable.

## Discussion

In this case, the patient developed hypotension and cutaneous symptoms immediately after the second ^99m^Tc-tetrofosmin administration. The symptoms met the diagnostic criteria for anaphylaxis [6], which was considered to be due to the ^99m^Tc-tetrofosmin administration.

Several national reports have been published on adverse reactions in the nuclear medicine department after radiopharmaceutical administration, with a frequency of <1×10-4 diagnostic cases [7-9]. These low-prevalence events are probably due to the minute amount of material used in the formulation of radiopharmaceuticals, resulting in mild, transient events that require little or no treatment [10, 11]. Additionally, the physician’s apprehension regarding potential liability, a belief that there is little interest in this information, and concerns about the complexity of reporting may have resulted in the inadequate reporting of these adverse events [11-13].

Several reports on allergies to ^99m^Tc radiopharmaceuticals, including ^99m^Tc-labeled diphosphonates [10,14,15], colloids [16], and albumin [16], have been published. However, no case of anaphylactic shock caused by the ^99m^Tc-tetrofosmin, ^99m^Tc-MIBI, or ^201^Tl preparations used in myocardial perfusion scintigraphy has been reported.

Myocardial perfusion scintigraphy is often performed to evaluate myocardial ischemia, particularly in patients with renal dysfunction or contrast media allergies, as in the present case. Therefore, clinicians should be mindful that, although rare, anaphylaxis can occur during myocardial perfusion scintigraphy.

Furthermore, even in situations where the trigger for anaphylaxis is unclear, respiratory or circulatory failure, in addition to cutaneous or mucosal symptoms, should be considered indicative of anaphylaxis [17]. In the present case, rapid rehydration and the administration of hydrocortisone and hydroxyzine improved the patient’s symptoms. However, the more appropriate course of action would have been the immediate administration of adrenaline after the onset of symptoms.

## Conclusions

To the best of our knowledge, we report the first case of anaphylactic shock due to a ^99m^Tc-tetrofosmin preparation during myocardial perfusion scintigraphy. Although rare, we should note that anaphylaxis can occur during myocardial perfusion scintigraphy. Furthermore, clinicians must maintain their readiness to promptly treat anaphylaxis, as it can occur suddenly and unexpectedly in various situations.
